# A retrospective study comparing the outcomes and toxicities of intensity-modulated radiotherapy versus two-dimensional conventional radiotherapy for the treatment of children and adolescent nasopharyngeal carcinoma

**DOI:** 10.1007/s00432-017-2401-y

**Published:** 2017-03-25

**Authors:** Wen-Ze Qiu, Xing-Si Peng, Hai-Qun Xia, Pei-Yu Huang, Xiang Guo, Ka-Jia Cao

**Affiliations:** 1 Department of Nasopharyngeal Carcinoma, Sun Yat-sen University Cancer Center, Guangzhou, 510060 People’s Republic of China; 2 Collaborative Innovation Center for Cancer Medicine, State Key Laboratory of Oncology in South China, Guangzhou, People’s Republic of China

**Keywords:** Nasopharyngeal carcinoma, Children and adolescents, Two-dimensional conventional radiotherapy, Intensity-modulated radiotherapy, Treatment result

## Abstract

**Purpose:**

To compare the clinical outcomes and toxicities of two-dimensional conventional radiotherapy (2D-CRT) and intensity-modulated radiotherapy (IMRT) for the treatment of children and adolescent nasopharyngeal carcinoma (NPC).

**Methods:**

A total of 176 children with non-metastatic NPC treated at Sun Yat-sen University Cancer Center between October 2003 and September 2013 were included in this study. Of the 176 patients, 74 received 2D-CRT and 102 were treated with IMRT. The clinical outcomes and acute and late toxicities were determined and compared.

**Results:**

The IMRT group achieved significantly higher overall survival (OS) (90.4% vs. 76.1% at 5 year, *P* = 0.007) and disease-free survival (DFS) (85.7% vs. 71.2%, *P* = 0.029) mainly due to an improvement in locoregional relapse-free survival (LRRFS) (97.9 vs. 88.3%, *P* = 0.049). After stratification by disease stage, IMRT provided significant benefits for patients with stage III–IV disease in terms of OS, LRRFS and DFS. Multivariate analyses indicated that the treatment group (2D-CRT vs. IMRT) was a prognostic factor for OS, LRRFS and DFS. A significant reduction in Grade 2–4 xerostomia (52.7 vs. 34.3%, *P* = 0.015) and hearing loss (40.5 vs. 22.5%, *P* = 0.010) was observed in patients treated by IMRT.

**Conclusion:**

IMRT provides better locoregional relapse-free survival and overall survival, especially in late-stage children and adolescent NPC patients, and is associated with a lower incidence of Grade 2–4 xerostomia as well as hearing loss compared with 2D-CRT. Distant metastasis remains a challenge in the treatment of children and adolescent NPC.

## Background

Nasopharyngeal carcinoma (NPC) is endemic in Southeast Asian countries, where the incidence rates range from 30 to 50 in 100,000 people. NPC is strongly associated with Chinese ethnic origin (Wei and Sham 2005; Wei et al. 2014). In most other populations, the disease is rare, with an intermediate incidence in Mediterranean basin countries and Greenland (Wei and Sham 2005). One remarkable difference between Mediterranean and Asian NPCs is the age distribution. Although a unimodal peak at 50–60 years exists among endemic countries, an additional minor early peak appears at 10–20 years of age among Mediterranean countries (Ayan et al. 2003). NPC in children differs from that of their adult counterparts in its close association with Epstein–Barr virus (EBV) infection, its undifferentiated histology, and the high incidence of locoregionally advanced disease (Ayan et al. 2003; Young and Dawson 2014).

The optimal treatment modality in children has not been established to date. The treatment recommendations for childhood NPC typically follow guidelines established for adults. Previous studies have reported that in paediatric and adolescent NPC patients treated with two-dimensional conventional radiotherapy (2D-CRT), the 5-year overall survival rate varied from 55 to 80%, with an incidence of late sequelae of 65–85% (Wolden et al. 2000; Hu et al. 2013).

Intensity-modulated radiotherapy (IMRT) is an innovative modality with the overall goal of providing a high dose of radiation to the tumour bed to achieve local control while attempting to spare the critical surrounding structures from high-dose radiation damage (Kwong et al. 2004). According to experiences with the use of IMRT for adults with NPC (Peng et al. 2012; Mao et al. 2015), it is expected that implementation of IMRT for children and adolescent NPC would also translate into an improved outcome with reduced treatment-related toxicities. However, studies that examine the application of IMRT in children and adolescent NPC are rare (Laskar et al. 2008; Guo et al. 2015), and no evidence has demonstrated significant advantages of IMRT compared with 2D-CRT regarding the survival rate and late treatment-related toxicities. The purpose of this retrospective study is to assess whether the use of IMRT is associated with improved clinical outcomes and reduced treatment-related toxicities compared with 2D-CRT in children and adolescent NPC.

## Methods

### Patient population

A total of 176 children with NPC were treated at the Sun Yat-sen University Cancer Center from October 2003 to September 2013. Patients were 7–20 years of age and histologically diagnosed with untreated non-metastatic NPC. Of the 176 patients, 129 were boys and 47 were girls (male/female ratio, 2.74:1). Most patients (69, 39.2%) presented with cervical lymphadenopathy. Other common presenting symptoms included epistaxis (35, 19.9%), tinnitus (28, 15.9%), headache (21, 11.9%), nasal obstruction or discharge (20, 11.4%), trismus (2, 1.1%) and facial numbness (1, 0.6%). Pretreatment evaluations included a complete history and physical examination, endoscopy and biopsy, complete blood count determination, liver and renal function tests, chest X-ray, and computed tomography (CT) or magnetic resonance imaging (MRI) of the head-and-neck region. CT scans of the abdominopelvis or chest, bone scans, and positron emission tomography scans were performed when clinically indicated. Patients underwent clinical staging according to the American Joint Committee on Cancer staging system (seventh edition). The medical ethics committee of Sun Yat-Sen University Cancer Center approved the study. All patients were treated under the principles of the Helsinki declaration.

### Treatment methods

#### 2D-CRT

The details of the 2D-CRT techniques utilized in our cancer centre were previously reported (Lai et al. 2011). Patients were immobilized in the supine position with a thermoplastic mask and treated with two lateral opposing faciocervical portals to irradiate the nasopharynx and upper neck in one volume followed by application of the shrinking-field technique to limit irradiation of the spinal cord. An anterior cervical field was used to treat the neck with a laryngeal block. The accumulated radiation doses were 66–80 Gy (median, 70 Gy), with 2 Gy per fraction applied to the primary tumour, 60–64 Gy applied to the involved areas of the neck, and 50 Gy applied to the uninvolved areas. All patients were treated with one fraction daily for 5 days per week.

A boost portal was performed if necessary. Different radiation energies, including megavoltage photons (6 or 8 MV) and electrons, were used. A boost dose (8 to 12 Gy per 4–6 fractions) was delivered to the skull base in patients with NPC involving the skull base and intracranial extension.

#### IMRT

The IMRT technique has also been described previously (Zhang et al. 2015). All patients were immobilized in the supine position with a head, neck, and shoulder thermoplastic mask. Two sets of images with and without contrast were obtained from the computed tomography (CT) simulator for treatment planning. All patients were scanned with serial 3-mm slices from the vertex through the clavicles. The gross tumour volumes of the nasopharynx (GTVnx) and positive neck lymph nodes (GTVnd) were delineated according to our previously described institutional treatment protocol (Zhao et al. 2004), which is in agreement with the International Commission on Radiation Units and Measurements Reports 50 and 62. The first clinical tumour volume (CTV1) was defined as the GTVnx plus a margin of 5–10 mm for potential microscopic spread, including the entire nasopharyngeal mucosa plus a 5-mm submucosal volume. The second CTV (CTV2) was defined by adding a margin of 5–10 mm to CTV1 and included the following regions, which required prophylactic irradiation: the retropharyngeal lymphnode regions, clivus, skull base, pterygoid fossae, parapharyngeal space, inferior sphenoid sinus, posterior edge of the nasal cavity, maxillary sinuses, and lymphatic drainage area. The planning target volume (PTV) for GTVs and CTVs were generated automatically by adding a 5-mm margin after delineation of tumour targets according to the immobilization and localization uncertainties. The prescribed dose was 62–70Gy (median, 68 Gy) to the PTV of the GTVnx (PTVnx), 58–66 Gy to the PTV of the GTVnd (PTVnd), 56–64 Gy to the PTV of the CTV1 (PTV1), and 50–58 Gy to the PTV of the CTV2 (PTV2) in 28–33 fractions. All patients were treated with one fraction daily over 5 days per week. The doses limited to the major organs at risk were as follows: the brain stem, with a 3-mm margin, *D*_max_ < 54 Gy; spinal cord, with a 5-mm margin, *D*_max_ < 40 Gy; the optic nerve, chiasm and temporal lobe, *D*_max_ < 54 Gy; the parotid gland, V30–35 < 50%.

#### Chemotherapy

In total, 166 (94.3%) patients received chemotherapy, with 126 and 117 patients treated by neoadjuvant and concurrent chemotherapy, respectively. Neoadjuvant chemotherapy consisted of a cisplatin and 5-fluorouracil (5-FU)-based regimen (PF regimen) every 3 weeks for 1–3 cycles. In concurrent chemotherapy, 23 patients received a 30 mg/m^2^/week cisplatin regimen (median cycles: 5) and 94 patients received 80 to 100 mg/m^2^/q3w cisplatin regimen (median cycles: 2).

#### Follow-up

Patients received follow-up every 3 months for the first 3 years after RT, every 6 months for years 3–5 and annually thereafter. Follow-up included physical examinations, chest X-ray, abdominal ultrasonography, MRI of the head and neck and/or bone scan. Late therapy-related complications were scored according to the Common Toxicity Criteria (CTC) version 3.0 of the U.S. National Institutes of Health. The last follow-up date was December 31, 2015.

### Statistical analysis

The following endpoints were assessed: overall survival (OS), locoregional relapse-free survival (LRRFS), distant metastasis-free survival (DMFS), and disease-free survival (DFS). OS was defined as the time from the start of RT to death by any cause. LRRFS was defined as the time from the start of RT to the first occurrence of local or regional recurrence. DMFS was defined as the time from the start of RT to the first occurrence of distant metastasis. DFS was defined as the time from the start of RT to the first occurrence of local or regional recurrence or distant metastasis. Pearson’s *χ* ^2^ tests were used to explore the differences between categorical variables. The actuarial rates were generated according to the methods of Kaplan and Meier. Differences between survival curves were compared using the log-rank test. Multivariate analysis was conducted by the Cox proportional hazard regression model. A *P* value of less than 0.05 was considered significant. Statistical analysis was performed using SPSS software, version 20.0 (SPSS, Chicago, IL).

## Results

### Patients’ clinicopathological characteristics

The characteristics of patients in the IMRT group and 2D-CRTgroup are presented in Table 1. A total of 102 patients received IMRT, and 74 received 2D-CRT. The groups were comparable with respect to the host factors, pathologic type, disease stage, and the use of chemotherapy (all, *P* > 0.05). The median follow-up time was 52 months (range 3–136 months).

**Table 1:** Characteristics of the 176 patients with paediatric and adolescent nasopharyngeal carcinoma

Characteristics	2D-CRT (%, *n* = 74)	IMRT (%, *n* = 102)	*P*
Gender			0.793
Male	55 (74.3)	74 (72.5)	
Female	19 (25.7)	28 (27.5)	
Age (years)			0.190
≤14	14 (18.9)	28 (27.5)	
>14	60 (81.1)	74 (72.5)	
Pathologic type			0.369
WHO II	0 (0)	3 (2.9)	
WHO III	74 (100.0)	99 (97.1)	
T stage			0.989
T1	2 (2.7)	3 (2.9)	
T2	2 (2.7)	7 (6.9)	
T3	37 (50.0)	44 (43.1)	
T4	33 (44.6)	48 (47.1)	
N stage			0.861
N0	5 (6.8)	6 (5.9)	
N1	21 (28.4)	29 (28.4)	
N2	31 (41.9)	48 (47.1)	
N3	17 (23.0)	19 (18.6)	
Overall stage			0.823
I	1 (1.4)	1 (1.0)	
II	1 (1.4)	4 (3.9)	
III	32 (43.2)	39 (38.2)	
IV	40 (54.1)	58 (56.9)	
Combination with chemotherapy			0.130
No	7 (9.5)	3 (2.9)	
NAC	33 (44.6)	16 (15.7)	
CCT	15 (20.3)	25 (24.5)	
NAC + CCT	19 (25.7)	58 (56.9)	

*2D*-*CRT* two-dimensional conventional radiotherapy, *IMRT* intensity-modulated radiotherapy, *NAC* neoadjuvant chemotherapy, *CCT* concurrent chemotherapy

### Patterns of treatment failure

Thirty-four patients (19.3%) failed during follow-up, including nine in locoregional relapse, 23 in distant metastasis and two in both (Table 2). The median failure times were 13 months (3–86 months) and 14 months (3–77 months) for locoregional relapse and distant metastasis, respectively. At the latest follow-up, 18 patients in the 2D-CRT group and 6 in the IMRT group died. The cause of death was related to recurrent cancer in 17 patients (94.4%) in the 2D-CRT group and 6 patients (100%) in the IMRT group.

**Table 2:** Patterns of disease failure in patients treated with 2D-CRT vs. IMRT

Failure pattern	2D-CRT [cases (%)]	IMRT [cases (%)]	*P*
Locoregional only	7 (9.5)	2 (2.0)	0.060
Distant metastasis only	13 (17.6)	10 (9.8)	0.131
Both locoregional relapse and distant metastasis	1 (1.4)	1 (1.0)	1.000

*2D*-*CRT* two-dimensional conventional radiotherapy, *IMRT* intensity-modulated radiotherapy

### Survival analysis

#### OS

**Fig. 1:**
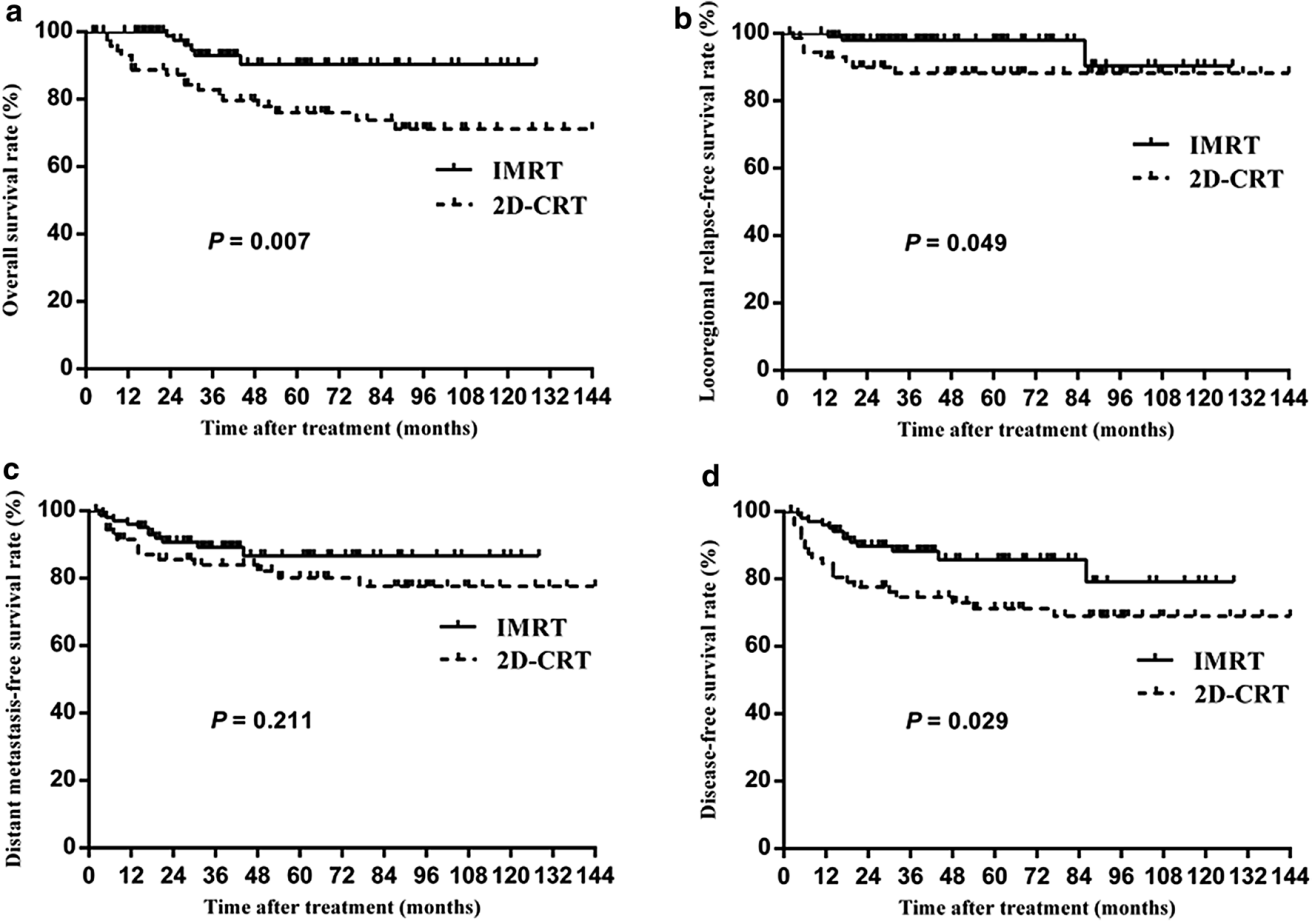
Comparisons of the overall survival (**a**), locoregional relapse-free survival (**b**), distant metastasis-free survival (**c**) and disease-free survival (**d**) for children and adolescent nasopharyngeal carcinoma (NPC) treated by two-dimensional conventional radiotherapy (2D-CRT) versus intensity-modulated radiotherapy (IMRT)

Figure 1a shows OS curves for patients in both study groups. At 5 years, the OS rates were 76.1% for the 2D-CRT group and 90.4% for the IMRT group. The difference was statistically significant [Hazard Ratio (HR): 0.30; 95% confidence interval (CI): 0.12–0.78; *P* = 0.007]. After stratification for the overall stage, the 5-year OS for stage III–IV disease for the IMRT group was increased compared to that of the 2D-CRT group (*P* = 0.011). After stratification for the T stage and N stage, the OS at 5 years was significantly greater for the IMRT group for patients with T3–4 or N2–3 disease (*P* = 0.021, *P* = 0.025, respectively).

#### LRRFS

Figure 1b presents the LRRFS curves for the patients in both study groups. A significant difference in LRRFS was observed between the groups (HR: 0.28; 95% CI: 0.07–0.98; *P* = 0.049). The LRRFS rates at 5 years were 88.3% in the 2D-CRT group and 97.9% in the IMRT group. The results of stratification for the overall stage revealed a borderline significant difference in LRRFS, favouring treatment with IMRT in patients with stage III–IV disease (HR: 0.29; 95% CI: 0.08–1.10; *P* = 0.052). After stratification for the T stage and N stage, no significant difference in LRRFS was observed between the groups.

#### DMFS

Figure 1c shows the DMFS curves for the study groups. There was no significant difference between the groups (HR: 0.61; 95% CI: 0.27–1.34; *P* = 0.211). The 5-year DMFS rates were 80.1% in the 2D-CRT group and 86.6% in the IMRT group. After stratification for the overall stage, T stage and N stage, no significant difference in DMFS was observed between the groups.

#### DFS

Figure 1d shows DFS curves for patients in both study groups. At 5 years, the DFS rates were 71.2% for the 2D-CRT group and 85.7% for the IMRT group. This difference was statistically significant (HR: 0.47; 95% CI: 0.23–0.94; *P* = 0.029). After stratification for the overall stage and N stage, the DFS at 5 years was significantly greater for the IMRT group for patients with stage III–IV or N2–3 disease (*P* = 0.037, *P* = 0.034, respectively). After stratification for the T stage, no significant difference in DFS was observed between the groups.

#### Prognostic factors

The value of various potential prognostic factors on predicting OS, LRRFS, DMFS, and DFS was evaluated. Univariate analysis by log-rank test showed that the N stage and treatment group were significantly associated with OS. The treatment group was significantly associated with LRRFS. Age, T stage, N stage, and overall stage were significantly related to DMFS. The T stage, N stage, overall stage and treatment group were significantly related to DFS (Table 3).

**Table 3:** Univariate analysis of prognostic factors for paediatric and adolescent nasopharyngeal carcinoma

Variate	5-year survival rate (%)							
	OS	*P*	LRRFS	*P*	DMFS	*P*	DFS	*P*
Gender		0.535		0.533		0.436		0.403
Male	81.5		93.2		82.2		77.7	
Female	89.9		95.3		87.5		83.2	
Age		0.886		0.292		0.023		0.243
≤14	88.0		89.1		97.6		86.7	
>14	82.7		95.2		79.9		77.2	
T stage		0.166		0.192		0.004		0.004
T1–3	88.5		95.6		90.8		86.7	
T4	78.1		91.6		75.4		70.6	
N stage		<0.001		0.079		<0.001		<0.001
N0–2	88.8		95.2		88.8		84.4	
N3	62.3		87.6		61.8		57.8	
Overall stage		0.212		0.197		0.022		0.015
Stage I–III	85.4		96.0		90.2		86.4	
Stage IV	79.7		92.0		78.4		73.5	
Treatment group		0.007		0.049		0.211		0.029
2D-CRT	76.1		88.3		80.1		71.2	
IMRT	90.4		97.9		86.6		85.7	
Chemotherapy		0.492		0.356		0.167		0.102
No	100		100		100		100	
Yes	82.5		93.4		82.5		77.9	
Neoadjuvant chemotherapy		0.686		0.532		0.955		0.590
No	83.1		95.0		83.2		80.2	
Yes	84.2		90.9		85.3		77.1	
Concurrent chemotherapy		0.472		0.748		0.507		0.338
No	85.3		94.6		84.4		80.1	
Yes	78.4		94.8		81.1		76.4	

*2D*-*CRT* two-dimensional conventional radiotherapy, *IMRT* intensity-modulated radiotherapy, *OS* overall survival, *LRRFS* locoregional relapse-free survival, *DMFS* distant metastasis-free survival, *DFS* disease-free survival

Multivariate analysis by Cox proportional-hazards model revealed that the T stage, N stage, overall stage and treatment group were independent prognostic predictors of OS (*P* = 0.017, *P* < 0.001, *P* = 0.031, *P* = 0.007, respectively). The treatment group was the only independent prognostic predictor of LRRFS (*P* = 0.041). The T stage and N stage were independent prognostic predictors of DMFS (*P* = 0.012, *P* < 0.001, respectively). The T stage, N stage and treatment group were independent prognostic predictors of DFS (*P* = 0.005, *P* < 0.001, *P* = 0.031, respectively) (Table 4).

**Table 4:** Multivariate analysis of prognostic factors for paediatric and adolescent nasopharyngeal carcinoma

Variate	OS		LRRFS		DMFS		DFS	
	HR (95% CI)	*P*	HR (95% CI)	*P*	HR (95% CI)	*P*	HR (95% CI)	*P*
Gender								
Male vs. female	1.12 (0.40–3.15)	0.834	0.88 (0.18–4.22)	0.868	0.94 (0.34–2.58)	0.901	0.97 (0.41–2.28)	0.942
Age								
≤14 vs. >14	0.61 (0.22–1.72)	0.348	0.38 (0.10–1.36)	0.135	5.55 (0.74–41.49)	0.095	1.33 (0.50–3.50)	0.568
T stage								
T1-3 vs. T4	6.66 (1.40–31.70)	0.017	3.53 (0.35–35.35)	0.284	14.14 (1.81-110.62)	0.012	8.63 (1.91–39.09)	0.005
N stage								
N0-2 vs. N3	11.94 (3.75–37.98)	<0.001	4.37 (0.89–21.38)	0.069	7.84 (2.97–20.64)	<0.001	6.41 (2.74–15.02)	<0.001
Overall stage								
I–III vs. IV	0.12 (0.02–0.83)	0.031	0.47 (0.03–7.36)	0.591	0.11 (0.01–1.15)	0.066	0.19 (0.03–1.09)	0.062
Treatment group								
2D-CRT vs. IMRT	0.27 (0.10–0.69)	0.007	0.24 (0.06–0.94)	0.041	0.65 (0.29–1.46)	0.299	0.46 (0.23–0.93)	0.031

*2D*-*CRT* two-dimensional conventional radiotherapy, *IMRT* intensity-modulated radiotherapy, *OS* overall survival, *LRRFS* locoregional relapse-free survival, *DMFS* distant metastasis-free survival, *DFS* disease-free survival

#### Acute and late toxicities

No significant differences in acute Grade 3–4 toxicities were observed between the two groups (Table 5). With respect to late complications, the rates of Grade 2–4 xerostomia and hearing loss were significantly greater in the 2D-CRT group compared with the IMRT group (Table 6). Of note, one patient in the 2D-CRT group died of severe radiation encephalopathy after 88 months.

**Table 5:** Acute Grade 3–4 toxicities in patients treated with 2D-CRT versus IMRT

Acute toxicity	2D-CRT (*n* = 74, %)			IMRT (*n* = 102, %)			*P*
	Grade 3	Grade 4	All	Grade 3	Grade 4	All	
Leucopenia	4 (5.4)	1 (1.4)	5 (6.8)	13 (12.7)	0	13 (12.7)	0.196
Neutropenia	5 (6.8)	1 (1.4)	6 (8.2)	11 (10.8)	5 (4.9)	16 (15.8)	0.133
Anaemia	2 (2.7)	0	2 (2.7)	2 (2.0)	0	2 (2.0)	1.000
Thrombocytopenia	0	1 (1.4)	1 (1.4)	3 (2.9)	0	3 (2.9)	0.640
Hepatotoxicity	1 (1.4)	0	1 (1.4)	0	0	0	0.420
Nephrotoxicity	1 (1.4)	0	1 (1.4)	1 (1.0)	1 (1.0)	2 (2.0)	1.000
Dermatitis	6 (8.1)	0	6 (8.1)	3 (2.9)	0	3 (2.9)	0.234
Stomatitis	16 (21.6)	0	16 (21.6)	18 (17.6)	0	18 (17.6)	0.510
Hearing loss	1 (1.4)	0	1 (1.4)	0	0	0	0.420
Total any	28 (37.8)	1 (1.4)	29 (39.2)	40 (39.2)	6 (5.9)	46 (45.1)	0.434

*2D*-*CRT* two-dimensional conventional radiotherapy, *IMRT* intensity-modulated radiotherapy

**Table 6:** Late toxicities in patients treated with 2D-CRT versus IMRT

Late toxicity	2D-CRT (*n* = 74, %)	IMRT (*n* = 102, %)	*P*
Xerostomia^a^	39 (52.7)	35 (34.3)	0.015
Hearing loss^a^	30 (40.5)	23 (22.5)	0.010
Skin dystrophy	29 (39.2)	30 (29.4)	0.175
Neck fibrosis	25 (33.8)	22 (21.6)	0.071
Trismus	11 (14.9)	8 (7.8)	0.138
Radiation encephalopathy	6 (8.1)	4 (3.9)	0.236
Cranial nerve palsy	7 (9.5)	4 (3.9)	0.134
Growth retardation	3 (4.1)	2 (2.0)	0.409

*2D*-*CRT* two-dimensional conventional radiotherapy, *IMRT* intensity-modulated radiotherapy

^a^Grade 2–4 toxicities

## Discussion

IMRT is an ideal radiation modality for NPC given its potential for excellent target coverage and normal tissue sparing. Several published meta-analyses are encouraging, demonstrating that IMRT not only improves locoregional control and survival but also reduces acute and late toxicities (Penagaricano and Papanikolaou 2003, 2012). However, because children and adolescents with NPC who meet the strict selection criteria accounted for only a small part of these studies, the real role of IMRT in this population remains unclear. Our current study aimed to assess whether the use of IMRT is associated with improved clinical outcomes and reduced treatment-related toxicities compared with 2D-CRT in children and adolescent NPC.

The results of this retrospective study comparing 2D-CRT with IMRT for the treatment of children and adolescent NPC showed that IMRT was associated with improved locoregional control and overall survival, especially for late-stage, non-metastatic disease, and a lower incidence of late toxicities.

In our study, improvement of locoregional control in the IMRT group for stage I/II patients was not remarkable. We hypothesize that the reason for this may be that 2D-CRT provides excellent locoregional control in early-stage NPC patients. However, the sample size of stage I/II (2 patients for 2D-CRT group and 5 for IMRT group) is insufficient to be statistically representative. Our results showed that the locoregional control rate was marginally significantly greater for non-metastatic stage III, and stage IVa and IVb patients in the IMRT group compared with the 2D-CRT group (*P* = 0.052). The tumour may extend close to the adjacent critical structures in patients with locoregionally advanced NPC, and the 2D-CRT dose and coverage often have to be compromised to avoid unacceptable complications, resulting in poor disease control. The aim of treatment for locoregionally advanced disease is to improve locoregional control by escalating the radiation dose in the tumour without exceeding the tolerance of the adjacent critical structures. IMRT can deliver a higher total dose and dose per fraction to the target volume and maintain low doses to the adjacent critical structures (Laskar et al. 2008). A prospective, randomized study by Peng et al. compared the outcomes and toxicities of IMRT vs. 2D-CRT for the treatment of NPC, and the result showed that better local recurrence-free survival and a lower incidence of toxicities can be achieved in the IMRT group for late-stage NPC patients (Peng et al. 2012). In the current study, the NPC failure pattern was not altered by IMRT, and distant metastasis remained the major pattern of failure in paediatric and adolescent NPC, a result similar to the findings of other reports (Tao et al. 2013; Guo et al. 2015). The relatively short time to systemic failure in the absence of locoregional failure might suggest that subclinical metastasis that likely existed at first treatment required early systemic treatment. Two prospective multicentre studies, NPC-91-GPOH (Mertens et al. 2005) and NPC-2003-GPOH/DCOG (Buehrlen et al. 2012), demonstrated favourable results for multimodal treatment (neoadjuvant chemotherapy, and radiotherapy followed by interferon-β) in patients with paediatric and adolescent NPC. After a median follow-up time of 30 months and 48 months, the overall survival and event-free survival rates were 95–97% and 91–92.4%, respectively. These promising outcomes indicate that interferon-β may provide a survival benefit in patients with paediatric NPC. However, further studies are needed to investigate the most effective chemotherapy regimens and their optimal timing with IMRT.

With better outcomes and longer survival, the prevention of radiation-induced complications becomes more important, and several studies have demonstrated quality of life advantages with IMRT in children with NPC (Tao et al. 2013; Guo et al. 2015). Laskar et al. (2008) compared 2D-CRT with IMRT in 36 cases of paediatric and adolescent NPC. A significant reduction in acute Grade 3 toxicities of the skin, mucous membrane, and pharynx was noted with the use of IMRT despite similar survival rates between the two groups. Moreover, the median time to the development of Grade 2 toxicities, including skin toxicity and mucositis, was delayed with IMRT. Tao et al. (2013) assigned 34 children with non-disseminated NPC to evaluate the long-term outcome and late toxicities with simultaneous integrated boost-intensity-modulated radiotherapy (SIB-IMRT). Grades 1–2 xerostomia and ototoxicity were the most common late toxicities. Only two patients (8.3%) developed grade 3 ototoxicity, and no patients developed grade 4 toxicities. The 5-year LRRFS, DMFS, DFS, and OS were 97.1, 88.2, 85.3, and 88.2%, respectively, which were comparable to the present data, confirming that an improved outcome and reduced toxicity could be achieved by IMRT in young NPC patients. Liu et al. (2014) studied 158 childhood and adolescence NPC patients to compare IMRT with 2D-CRT. The result revealed that IMRT significantly reduces trismus (27.3 vs. 3.6%, *P* = 0.03) and Grade 2 xerostomia (37.9 vs. 10.3%, *P* = 0.02); however, no survival benefits are achieved with the use of IMRT. Similarly, our study reported a significant reduction of Grade 2–4 xerostomia and hearing loss (*P* = 0.015, *P* = 0.010, respectively) using IMRT.

As one of the most feared complications after radical radiotherapy, temporal lobe injury (TLI) can be devastating for patients and accounts for approximately 65% of deaths from radiation-induced toxicities (Lee et al. 1992). The results from our study revealed a trend of higher incidence of radiation encephalopathy in 2D-CRT compared with IMRT (8.1 vs. 3.9%, *P* = 0.236), and one patient in the 2D-CRT group died of severe radiation encephalopathy in 88 months. Zhou et al. (2013) reported that IMRT significantly reduced the incidence of radiation-induced TLI compared with the 2D-CRT (7.5 vs. 10.8%, *P* = 0.048). Mao et al. (2014) also observed less extensive and milder temporal lobe necrosis (TLN) in the IMRT group. We hypothesize that the improvement observed with IMRT is largely due to the technical advantages of IMRT. Compared with opposed lateral fields in conventional 2D radiation therapy, modern IMRT utilizes multiple small segments of beams (pencil beams), and the intensities of the neighbouring pencil beams vary. Collectively, beams composed of segments with different intensities produce dose distributions that conform to the required shape of the targets. Furthermore, the popular adopted simultaneous integrated boost (SIB) dosing regimen has the advantage of providing a more conformal dose distribution, thereby facilitating enhanced sparing of critical normal structures (Chen et al. 2005).

To the best of our knowledge, the present analysis is the only study reporting significant advantages of IMRT compared with 2D-CRT regarding the survival rate and late treatment-related toxicities in children and adolescent NPC. Our study, however, has several limitations. The main limitation is its retrospective nature and the sample size. The establishment of treatment guidelines for paediatric NPC is needed through multi-centre collaboration on randomized controlled trials. Furthermore, radiation-induced toxicities may develop over time, especially for young patients, and a longer follow-up period is desirable to determine the exact incidences of these complications and the actual superiority of IMRT in reducing late toxicities (e.g. endocrinopathies, cranial nerve palsy, and second neoplasms).

In conclusion, IMRT provides better locoregional relapse-free survival and overall survival, especially in late-stage children and adolescent NPC patients and is associated with a lower incidence of Grade 2–4 xerostomia and hearing loss compared with 2D-CRT. Distant metastasis remains a challenge in the treatment of children and adolescent NPC.

## Abbreviations

2D-CRT: Two-dimensional conventional radiotherapy
IMRT: Intensity-modulated radiotherapy
SIB-IMRT: Simultaneous integrated boost intensity-modulated radiotherapy
NPC: Nasopharyngeal carcinoma
OS: Overall survival
DFS: Disease-free survival
LRRFS: Locoregional relapse-free survival
DMFS: Distant metastasis-free survival
LRC: Locoregional control
EBV: Epstein–Barr virus
CT: Computed tomography
MRI: Magnetic resonance imaging
GTV: Gross tumour volume
CTV: Clinical tumour volume
PTV: Planning target volume
CTC: Common Toxicity Criteria
TLI: Temporal lobe injury
TLN: Temporal lobe necrosis
NAC: Neoadjuvant chemotherapy
CCT: Concurrent chemotherapy
WHO: World Health Organization
